# The Engineering Side of Hospital Work

**Published:** 1905-02-04

**Authors:** 


### THE ENGINEERING SIDE OF HOSPITAL WORK.

*Continued from page 322.*

### II.—COLD STORAGE AND REFRIGERATION.

In dealing with the several subjects enumerated in the previous article the writer proposes to give a *résumé* of the principles governing the different appliances, a short description of the leading and best known apparatus, and to follow with a description of the apparatus actually in use in each hospital.

### The Principles of Cold Storage.

Cold storage, as the name implies, means the provision of rooms, cabinets, or boxes, in which the temperature is maintained at a certain low figure, such as will arrest the decay of produce, the temperature being controlled by the engineer in charge of the apparatus. In order that this may be accomplished, the rooms or boxes must be so constructed that heat passes into them with difficulty, and that any heat which does pass in is absorbed, in addition to the heat of the atmosphere of the room when the refrigerating apparatus is first put in operation, and of the produce or other things that are required to be preserved.

The principles upon which the whole system depends are very simple.

1. All bodies allow heat to pass through them, but all bodies offer a certain resistance to its passage, and certain substances offer a very high resistance. Hence, if one of these substances is used in the building of the rooms, and the construction of the cabinets and boxes in which produce is to be kept, in such a manner that the heat passing into the room or box must pass through the substance, the quantity of heat passing into the room or box will be comparatively small.

2. All substances may exist in the solid, the liquid, or the gaseous condition, the state in which they are at any moment being ruled by the quantity of heat they have absorbed, their specific heats, and the pressure to which they are subject. Further, while the temperature of any body rises steadily, or falls steadily, as definite quantities of heat are absorbed by, or abstracted from it; when the physical condition is changed, as when the substance passes from the liquid to the gaseous, or from the solid to the liquid state, or *vice versâ*, large quantities of heat are absorbed, or liberated, without change of temperature. Thus, while 1 lb. of water requires, approximately, 1 B. Th. Unit to be delivered to it to raise its temperature 1° F., or to have 1 B. Th. Unit abstracted from it to lower its temperature by a similar amount, immediately boiling-point is reached, 966 B. Th. Units are required to convert the 1 lb. of water into steam without change of temperature, and 142 B. Th. Units must be abstracted from the same 1 lb. of water when it reaches freezing-point before it can all be frozen, and again without change of temperature. Certain substances, *e.g.* ammonia, carbonic acid, and sulphurous acid change their physical condition very readily from the liquid to the gaseous, and *vice versâ*, and these substances are employed and this property made use of to extract the heat from the cold chambers; while for cabinets or boxes, the latent heat of water and the heat abstracted by solution are made use of.

### The Cold Chamber.

The cold chamber, cabinet, or box in which substances are to be preserved may be of any convenient size, from the box in which a few head of dead poultry may be kept, to a large warehouse capable of storing several hundreds of sheep, quarters of beef, etc., but they are always constructed on certain definite lines. As mentioned above, some substances have the property of resisting what the writer has called heat-currents—the passage of heat through them, and the walls, roof, and floors of the cold chambers are all constructed so that there is a large mass of one of these substances through which the heat must pass on its way to the chamber. The substances that have been found best for thermal insulating purposes are those in which the mass of the substance is broken up very much, so that the heat, in passing through the substance, has to be continually changing from the substance to air and back to the substance. The idea has been that substances such as cork, silicate cotton (slag wool), finely-divided charcoal, which are those almost universally employed in all large work, and which enclose a number of minute air cells, owe their insulating properties to the presence of the dry air in the cells, it being the impression that dry air is one of the best thermal insulators known. There appears to be some doubt as to this, however, and it is more probable that the insulating property is due to the features mentioned, the continual change of the medium through which the heat passes. Similar phenomena are met with in electricity. In any case these substances, whatever the cause, offer the highest resistance known to heat currents. Sawdust, when quite dry, hair felt, wool, and other substances, all, it will be noticed, having the same characteristic features, the minute subdivision, and the consequent passage of the heat current from the substance to air and back again, are also sometimes used. The walls, roof, and floor of cold chambers are built hollow, so that as much of the insulating material as possible may be enclosed. One favourite form is an outer containing wall of match-boarding fixed to wooden supports, the inner side of the wood being covered with some kind of waterproof material to exclude moisture. A few inches from the outer wall, from 4 inches up to 11 inches, according to the other conditions, a second similar wall is fixed, supported by wood uprights, the two wooden walls being separated by wooden distance pieces. The outer face of the inner wooden wall is also covered with some waterproof material; there are papers specially made for this purpose, and the space between the two walls is filled in, as tightly as possible, with one of the substances named, for example, slag wool or charcoal in a very finely divided state. Cork is sometimes broken up for use, but in a few of the later installations, cork bricks have been employed with success. The air-cells mentioned, being in the cork, the result is the same. For the floor, asphalt is often used over the insulating material. The cold chamber is fitted with a door, exactly corresponding to the walls, and built up in the same way. It is made to close very tightly, so as to exclude all possibility of air from outside entering by the space between the door and its containing wall. It is much better, where it can be done, to fit two doors, forming an air lock. Where there are two adjoining cold chambers, it is usual to build a lobby, or vestibule, with three doors, one opening to the outer atmosphere, and one to each chamber. The attendant passes into the vestibule first, closing the outer door, then into either of the chambers. By this arrangement the ingress of outer air, when either chamber is opened, is reduced to a minimum. Without it a good deal of additional work is thrown on the refrigerating plant. Cold chambers are usually lighted by electric incandescent lamps, which are turned in and out from switches outside the chambers. Cabinets or boxes are constructed on exactly the same lines, the doors being only large enough to enable the attendants to handle the produce stored.

### Cooling the Cold Chambers or Boxes. Small Cabinets and Boxes.

For small cabinets, such as are used in hotels, in the serving rooms, and in hospitals for certain purposes, and for the small boxes that are made to take small quantities of produce, ice, or ice mixed with either common salt or carbonate of soda, or some other salt, provides sufficient cooling for the purpose. As mentioned above, in order that water may freeze 142 B.Th. Units must be abstracted from every 1 lb., and consequently when ice is placed in a position where it can receive heat, as in a cold cabinet or box, it gradually melts, each 1 lb. of ice absorbing the same number of B.Th. Units as were abstracted from the water when it was formed. Thus it is a very simple calculation, knowing the cubic capacity of the box, or cabinet, the specific heat of air, and the approximate rate at which heat will pass through into the box, or cabinet, to find how much ice is required to keep the temperature of the box below a certain figure for a certain time.

### The Action of Freezing Mixtures.

It was mentioned above that ice and common salt, or ice and carbonate of soda, or ice and other substances, can be used for small work in refrigeration. The mixtures named will be recognised as a few of the many freezing mixtures. They give additional effect to the ice in the following manner. Where one of the mixtures is employed, it is placed in the vessel in a crushed state, the pounded ice and the salt being thoroughly mixed together. As the ice melts the water so formed dissolves a portion of the salt with which the ice has been mixed. The solution of any substance, a solid in a liquid, for instance, is now recognised to be a definite physical action, in which the dissolved substance is raised to the liquid form, the heat required for the purpose being taken from the liquid in which the solution takes place, and its containing vessel, etc. Hence the solution of the salt, carbonate of soda, or other substance abstracts additional heat from the chamber, box, or cabinet in which it is enclosed. Small chambers, capable of storing larger quantities of produce than can be placed in cabinets, are worked on this plan. It is not as economical as the others that will be described, except for small work. Above a certain small-sized chamber, it would be cheaper to provide mechanical apparatus. The crucial point is, of course, the labour item. Mechanical apparatus requires attendance of a skilled kind, though makers claim that any shopman can easily attend to their apparatus; while the arrangement where ice, or ice and a salt are used, only requires filling and seeing that the box is properly closed. The usual arrangement with these small chambers, or cabinets which are worked by ice, or ice and a salt, is to place the ice in a galvanised iron tank, or in a cage with a tray underneath, to catch the water as it is formed. It is also usually arranged, where possible, that the air passes continually over the outside of the ice-box. This is generally easily arranged. The warm air rises, that portion of it that is in the neighbourhood of the ice-box passes over the outside, and convection currents are set up, leading to a more or less perfect exchange of the air, so that all parts of the chamber are kept at about the same temperature. With the boxes and cabinets that are used in hotel serving-rooms and similar places, the air is changed when the doors are open, the fresh air that enters the cabinet being carried up to the ice-box, cooled, and so on. In some small chambers where only ice, or ice and a salt are employed, a small fan is used, worked by any convenient source of power, as by a small electric motor, to keep the air in continual circulation within the chamber. As will be explained later, the circulation of the air is of very great importance in cold storage work, particularly as cooling the air leads to its being dried, the moisture depositing on the cold surfaces of the ice-box.

### Ice-making Tanks.

Tanks for making ice are constructed on very much the same lines, the tank in which the water to be frozen is carried being insulated in the manner described for cold chambers, except, of course, the top of the tank.

### Cooling Larger Chambers.

For larger work than that described, where considerable quantities of produce have to be stored, one of the methods mentioned above are employed, in which ammonia, carbonic acid, or sulphurous acid, are alternately liquefied by pressure and cooling, and allowed to return to the gaseous condition, the heat required for their expansion to the gaseous condition being taken, as fully as possible, from the air, or produce to be cooled. It will be understood that, in the small apparatus that have been described, the heat of the substances to be preserved, and that penetrating to the cold cabinet, or chamber, is absorbed by the ice, or ice and a salt. In the larger apparatus, the heat present in the cold chambers, from whatever cause, is transported to the cooling water that is employed in reducing the gas to the liquid condition, and in some cases is transferred from that to the atmosphere, thus completing the circuit, as electrical engineers would term it. In the next article the different methods of mechanically producing low temperatures will be described.

